# Assays to enhance metabolic phenotyping in the kidney

**DOI:** 10.1152/ajprenal.00232.2024

**Published:** 2025-01-17

**Authors:** Safaa Hammoud, Justin Kern, Sandip Mukherjee, Andrew J. Lutkewitte, Prabhleen Singh, Kate Newberry, Brian N. Finck, Leslie S. Gewin

**Affiliations:** 1 Division of Nephrology, Department of Medicine, Washington University in St. Louis, St. Louis, Missouri, United States; 2 Division of Nutritional Sciences and Obesity Medicine, Department of Medicine, Washington University in St. Louis, St. Louis, Missouri, United States; 3 Division of Nephrology and Hypertension, University of California San Diego, San Diego, California, United States; 4 Department of Medicine, VA San Diego Healthcare System, San Diego, California, United States; 5 Department of Medicine, Veterans Affairs Hospital, St. Louis VA Health Care System, St. Louis, Missouri, United States

**Keywords:** fatty acid oxidation, kidney metabolism, mitochondrial respiration, proximal tubules

## Abstract

The kidney is highly metabolically active, and injury induces changes in metabolism that can impact repair and fibrosis progression. Changes in the expression of metabolism-related genes and proteins provide valuable data, but functional metabolic assays are critical to confirm changes in metabolic activity. Stable isotope metabolomics is the gold standard, but these involve considerable cost and specialized expertise. Both the Seahorse bioflux assays and substrate oxidation assays in tissues ex vivo are two relatively cost-effective assays for interrogating metabolism. Many institutions have access to Seahorse bioflux analyzers, which can easily and quickly generate data, but guidelines to enhance reproducibility are lacking. We investigate how variables (e.g. primary vs. immortalized cells, time in culture) impact the data generated by Seahorse bioflux analyzers. In addition, we show the utility of ^3^H-palmitate, a new approach for assessing fatty acid oxidation (FAO) in the kidney, in uninjured and injured kidney cortices. The ^3^H-palmitate substrate oxidation assays also demonstrate significant sex-dependent and strain-dependent differences in rates of fatty acid oxidation. These data should facilitate metabolic interrogation in the kidney field with enhanced reproducibility.

## INTRODUCTION

The kidney is a highly metabolically active organ as considerable energy is required to support the transporters necessary to reabsorb more than 98% of filtered fluid and solutes (1). The tubules, particularly the proximal tubules, generate high amounts of ATP through mitochondrial respiration (2, 3). Changes in metabolism are being appreciated in both the acutely and chronically injured kidneys, and increasing attention is being paid to how these changes in metabolism alter responses to injury (4, 5). Investigators may realize that their protein of interest modulates metabolism and want to interrogate these metabolic changes further, but exploring metabolism in the kidney presents some challenges.

Entering the field of metabolism and choosing the proper tool or metabolic assay to answer a question can be daunting. Untargeted metabolomics, which offers a snapshot in time of a wide range of metabolites, can be extremely useful for generating a hypothesis or identifying a pathway that may be altered. However, assessment of steady-state metabolite concentrations is not necessarily indicative of metabolic flux, and these studies often generate large amounts of information that need expertise to interpret. The most sophisticated functional metabolic assays use stable isotope-labeled substrates that can provide more dynamic information about metabolic pathway flux. These stable isotope studies require expertise in both metabolism and bioinformatics and considerable expense, thus limiting their use for many investigators.

The Seahorse bioflux analyzer is commonly used to interrogate mitochondrial function and is widely available at Core facilities in many institutions. However, key limitations regarding the type of cells being used, time in culture, and details regarding normalization are often overlooked, potentially impacting reproducibility (6, 7). The metabolism of cultured cells can yield valuable information, but the artificial growth conditions make its relevance to kidney cells in vivo questionable. Thus, there is a need to interrogate metabolism in tissue without the expense and expertise required of stable isotope studies. The use of radioactive-labeled substrates to measure metabolic pathways in tissue ex vivo is a cost-effective and time-efficient approach. The use of ^3^H-palmitate (i.e., tritiated long-chain fatty acid) has been published in skeletal muscle tissue ex vivo (8). When ^3^H-palmitate undergoes β-oxidization, reducing equivalents (NADH and FADH_2_) generated from β-oxidation itself, and oxidation of acetylcoenzyme A (acetyl-CoA) in the tricarboxylic acid (TCA) cycle contain ^3^H. When these reducing elements donate electrons to the electron transport chain (ETC), ^3^H_2_O will be generated by complex IV. This ^3^H_2_O can be quantified. We optimized this method for kidney tissue and validated that in the unilateral ureteral obstruction (UUO) model, the classic model of tubulointerstitial fibrosis, fatty acid oxidation (FAO) is significantly reduced even at relatively early time points. Published literature has suggested that there may be sex-specific differences in metabolism (9), but this has not, to our knowledge, been tested with functional assays. Using ^3^H-palmitate, we observed that female mice have significantly higher rates of fatty acid oxidation compared with kidneys from males.

## MATERIALS AND METHODS

### Animal Model

Male FVB mice (Jackson Labs) 8–10 wk of age were used to isolate primary proximal tubule-enriched (PT) cells and fresh isolations of tubules ex vivo. Unilateral ureteral obstruction (UUO), a classic model of tubulointerstitial fibrosis, was performed as described previously (10). Briefly, the right kidney was exteriorized using a dorsal incision, and the ureter was ligated twice at the base of the renal pelvis. Muscle and skin layers were sutured closed, and mice were euthanized 2 or 5 days after UUO. To check for sex-specific differences, both male and female mice of FVB and C57BL/6J strains at 8–10 wk of age were used. All procedures were approved by the Institutional Animal Care and Use Committee of Washington University in St. Louis.

### Isolation of Fresh Primary Proximal Tubule-Enriched Cells

PT cells were generated from renal cortical tissue as previously described (11, 12). Briefly, renal cortical tissue was minced and digested at 37°C for 45 min in RPMI media (Corning, 15–040) containing collagenase I (1 mg/mL), dispase (1 mg/mL), and DNAse (Bio-Rad, 7326828, 1:100). The slurry was passed through a 70-μm filter and washed twice with RPMI by centrifugation at 300 *g* for 5 min. To remove red blood cells, a third centrifugation was performed at 18 *g* for 30 s and the supernatant was removed. Primary PT cells were grown in RPMI media with additives and supplements listed in Table 1, and fetal bovine serum (FBS) was reduced to 5% 3 days after plating. After 3 or 10 days in culture, cells were plated onto a 24-well or 96-well Seahorse plate at a density of 100,000 cells/well or 63,000 cells/well, respectively, using a substrate-limited media (SLM) prepared with DMEM (Corning, 7–207-CV) and additives listed in Table 2. On the following day, the SLM media was changed into Krebs–Henseleit buffer (KHB) (Table 3), and Seahorse bioflux assay was performed.

### Proximal Tubule Conditionally Immortalized Cells

PT conditionally immortalized cells (PT-immortalized), previously generated and characterized (13, 14), were grown at 33°C in DMEM/F12 media (Corning, 10–092-CV) with 2.5% FBS, PT supplements, and antibiotics listed in Table 1, and IFN-γ 10 ng/mL. Three days later, IFN-γ was removed, and the cells were transferred to the Seahorse plate (Agilent) in SLM and placed in 37°C with 5% CO_2_. The next day, SLM was changed into KHB media, and Seahorse bioflux assay was performed.

### Human Renal Proximal Tubule Epithelial Cells

Primary human renal proximal tubule epithelial cells (RPTECs) were obtained from Lonza (CC-2553) and cultured in renal epithelial cell growth basal media (REBM, Lonza, CC-3191) supplemented with renal epithelial cell growth media BulletKit (REGM, Lonza, CC-4127) according to the manufacturer’s protocol. Cryopreserved RPTEC are shipped in the first or second passage per Lonza. Upon receipt from Lonza, RPTECs were expanded and stored in liquid nitrogen. The day before the Seahorse bioflux assay, the RPTECs were thawed and directly plated onto a Seahorse plate in SLM. The next day, the media was changed to KHB media, and Seahorse bioflux assay was performed.

### Fresh Isolation of Tubules Ex Vivo

Fresh tubules were isolated from kidney cortices according to a method previously published (15–17) with modifications. Briefly, kidney cortical tissue was minced, placed in 10 mL of Hanks balanced salt solution (HBSS) containing collagenase type I (0.7 mg/mL), and incubated in a water bath at 37°C for 30 min. Tubules were mixed every 5 min by inverting the tubes and every 15 min by pipetting. After digestion, the 10-mL solution was split into two tubes (5 mL each) and 5 mL of heat-inactivated horse serum was added to the tubules and vortexed for 30 s. Tubules were allowed to sediment for exactly 1 min. The supernatant was then centrifuged at 200 *g* for 7 min. The supernatant was discarded, and the pellet was washed with HBSS and resuspended with 2-mL KHB, Table 3, for Mitostress and 2 mL XF Base Medium (Agilent 102353–100) with l-glutamine 2 mM per Agilent’s recommendations, for glycolysis. We optimized the concentration of tubules to plate (Supplemental Fig. S2, A–C) and, based on this, plated roughly 12.5 μg/mL per well from uninjured kidneys and 25 μg/mL from injured kidneys. The tubules were plated on the XFe96 cell culture plate (Agilent) that had been precoated with poly-d-lysine (50 μg/mL, Fisher A38904–01). The coating material was selected after testing several options including Cell-Tak, Matrigel, and collagen, and no significant differences were observed apart from bovine serum albumin (BSA), which showed a lower adhesion (Supplemental Fig. S2, E and F). After tubules were plated, the plates were centrifuged at 18 *g* for 5 min to augment adherence of the tubules. Cellular respiration and glycolytic function were then immediately measured by Seahorse bioflux analyzer (see Seahorse Bioflux Assay Normalization).

### Bioflux Analyses

**Figure 1.**
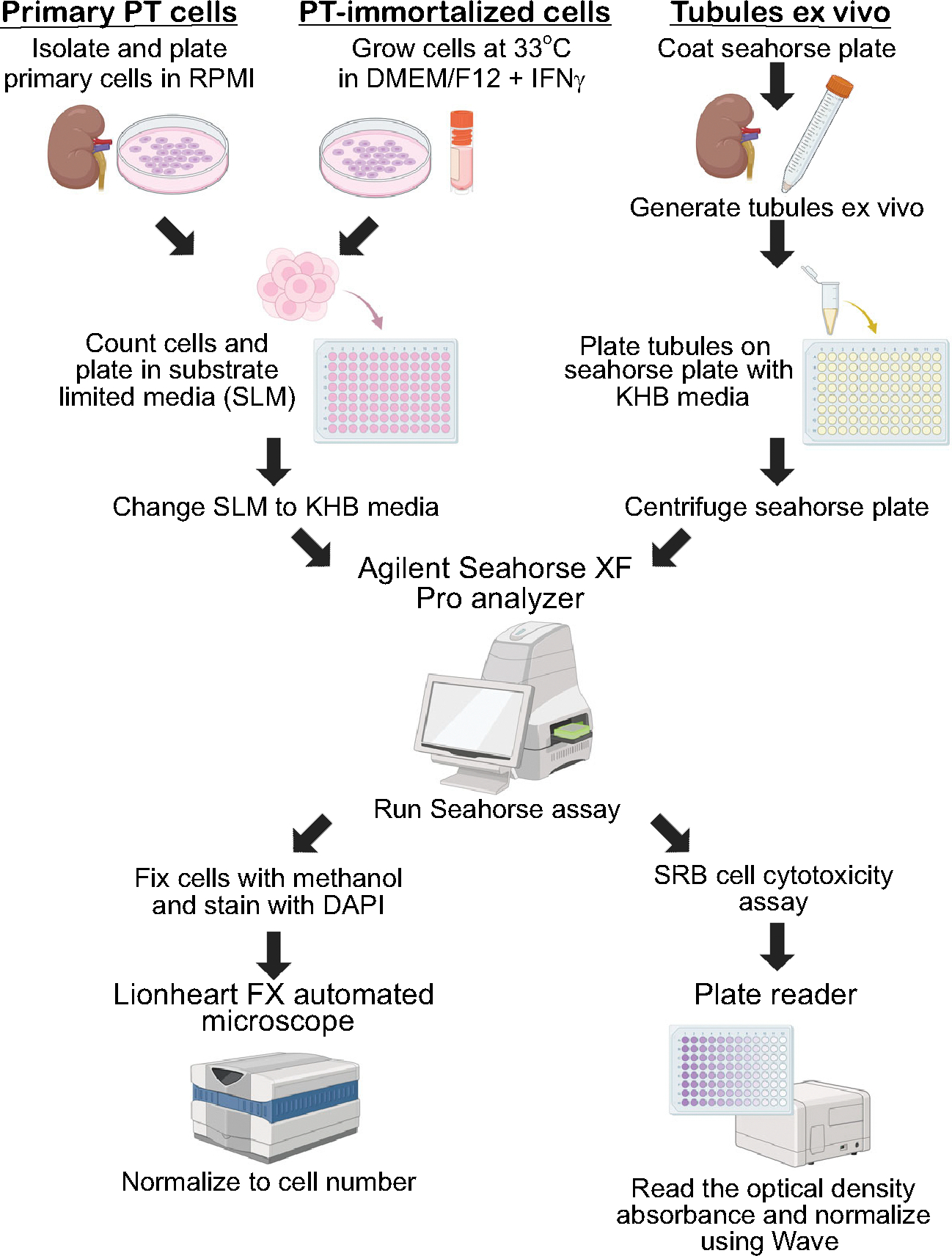
Workflow of preparing primary and immortalized PT cells and tubules ex vivo for Seahorse bioflux assay. Primary PT cells are initially grown in RPMI media, whereas PT conditionally immortalized cells (PT-immortalized) are grown in DMEM/F12 (see MATERIALS AND METHODS). The day before performing Seahorse bioflux analysis, both primary PT and PT-immortalized cells are trypsinized, counted, and replated in substrate-limited media (SLM, Table 2). It is critical to mix the cell suspension while plating to ensure uniform plating. After plating on the Seahorse plate, spin the plate down at 18 *g* for 1 min and incubate overnight at 37°C with 5% CO_2_. The Seahorse cartridge plate is hydrated overnight in a CO_2_-free incubator. The day of the assay, remove the SLM media from the wells by pipetting and replace it with KHB media (Table 3). When the Seahorse run is performed, fix the cells with 100% cold methanol, stain with DAPI, and count the cells using the Lionheart FX automated microscope (Biotek). Normalize the bioflux analyses assay results via cell count in each well using Wave software. For freshly prepared tubules ex vivo, precoat the Seahorse plate using poly-d-lysine (50 μg/mL in Dulbecco’s PBS). Generate the tubules as described (see MATERIALS AND METHODS). Based upon Supplemental Fig. S2, A and B, plate 12.5–25 μg/mL on the Seahorse plate with KHB media for Mitostress and XF Base Media (see MATERIALS AND METHODS) for glycolysis, mixing the tubules well throughout the plating process. After performing the Seahorse bioflux analysis, run the sulforhodamine B (SRB) cell cytotoxicity assay and measure optical density absorbance (OD). Normalize the bioflux analyses assay results to the OD obtained from SRB assay in each well using Wave software. KHB, Krebs–Henseleit buffer; PT, proximal tubules; SLM, substrate limited media; SRB, sulforhodamine B. Created in BioRender (https://BioRender.com/b09v445).

Oxygen consumption rate (OCR) and extracellular acidification rate (ECAR) of cells and kidney tubules were measured using Seahorse XFe24 or XFe96 extracellular flux analyzers (Agilent Technologies, Billerica, MA). The protocols used have been adopted from Agilent Seahorse protocols and modified as in Fig. 1. For mitochondrial function, we measured OCR through the Mito Stress assay using Oligomycin (3 μM), FCCP (2 μM for plated cells or 4 μM for freshly isolated tubules), and antimycin A/rotenone (2 μM) as previously described (16). Different numbers of plated cells, volumes of freshly isolated tubules, and concentrations of carbonyl cyanide 4-trifluoromethoxyphenylhydrazone (FCCP) were used to determine the optimal number/concentrations for this assay (Supplemental Figs. S1, A–C, and S2, A–D). To measure glycolysis, ECAR was assessed in response to glucose (10 mM), oligomycin (1 μM), and 2-deoxyglucose (2-DG, 50 mM), per Agilent’s glycolysis protocol.

### Seahorse Bioflux Assay Normalization

After metabolism was interrogated with Seahorse, the cells were fixed with methanol, stained with 4′6-diamidino-2-phenylindole (DAPI), and counted using the Lionheart FX automated microscope (Biotek). The bioflux analyses assay results were normalized via cell count in each well using Wave software. The number of cells per well was counted and averaged, and wells with a cell count ±15% of the average were omitted to minimize variability. For experiments performed on freshly isolated tubules ex vivo, the DAPI-based staining approach was not feasible. Instead, the sulforhodamine B (SRB) cell cytotoxicity assay kit (Biovision K943–1000) was used. We followed the SRB kit protocol to get the optical density (OD) absorbance.

### Radioactive Fatty Acid Oxidation Assay

Fatty acid oxidation (FAO) was assessed in tissue ex vivo by using ^3^H-palmitate and measuring the release of ^3^H_2_O (18–20). [9,10-^3^H] palmitate (PerkinElmer.; NET043001MC) was conjugated to fatty acid-free bovine serum albumin (BSA) (Sigma-Aldrich, A6003). To do this, BSA was dissolved in 13 mL of phosphate-buffered saline (PBS) (2.5 mg/mL). In a separate glass vial, unlabeled palmitate (Fluka, No. 76119) was dissolved in 1 mL of heated 95% ethanol at a concentration of 50 mM. Unlabeled palmitate (130 μL) and 100 μL ^3^H-palmitate (0.5 mCi) stock was mixed with the 13-mL BSA solution. The BSA/palmitate solution was vigorously vortexed and incubated at 37°C overnight with constant rotation at 50 rpm for complex formation. The final concentration of palmitate in this stock (RAD stock) was 500 μM (labeled and unlabeled). Conjugated ^3^H-palmitate/BSA was prepared in advance, aliquoted into smaller batches, and frozen at −20°C for use in multiple assays to diminish inter-assay variation due to batch effects.

One day before the assay, ion-exchange columns were prepared: the tips of glass Pasteur Pipets (Fisher, 13–678–20 D) were sealed by melting them with Bunsen burner flame and then plugged with 1 cm of fiberglass (Glass wool, VMR 32848–003). Sealed pipets were placed vertically (tip down) in a tube rack. Resin (AmberChrom 1X2 chloride form, 200–400 mesh, Sigma 217395) was prepared as a slurry (0.37 mg/mL resin in water) in a beaker with constant stirring. Approximately 2.4 mL of resin slurry was pipetted into each sealed pipet, air bubbles were carefully removed, and resin was left to settle overnight at 4°C (see Fig. 6 for a diagram of this workflow).

On the day of the assay, ~40 mg of kidney cortex was homogenized in 700-μL PBS with CaCl_2_ (1.2 mM) and MgCl_2_ (1 mM) (Sigma; D8662) using a Dounce homogenizer (Grainger, 23NE02). A 100-μL homogenized lysate was further diluted with 100-μL PBS (with calcium and magnesium) and used for the assay. The reaction mixture was prepared by diluting the above RAD stock (500 μM) with l-carnitine (stock 100 mM, Sigma, C0158) in PBS plus CaCl_2_/MgCl_2_ (Table 4). In the reaction mixture, the concentration of palmitate was 125 μM and l-carnitine was 0.99 mM. The volume of the reaction mixture prepared was based on the number of samples to be assayed. The reaction mixture was warmed at 37°C for 15 min before the assay. For the assay, 100 μL of diluted kidney lysate was placed in a 12-well plate, and 100 μL of the reaction mixture was added. Thus, the final palmitate concentration in the 200-μL assay was 62.5 μM. Negative controls (blanks) were prepared by mixing equal volumes of PBS and the reaction mixture without any kidney tissue. Samples were incubated on a rotating shaker (50 rpm) at 37°C in a cell culture incubator for 2 h. After incubation, 150 μL of supernatant liquid from each well and the blank (no kidney tissue control) were transferred to a tube containing 150 μL of 10% trichloroacetic acid (TCA) to stop the reaction. The tubes were vigorously vortexed and centrifuged for 10 min at 2,200 *g* at 4°C to pellet the precipitated unmetabolized palmitate, and the supernatant (250 μL) was transferred to a new tube and mixed with 46 μL of 6 M NaOH to neutralize the TCA (LabChem, LC262301). Remaining tissue homogenates from each well were collected, and protein content was determined by BCA assay.

The sealed tip of the ion-exchange columns was manually snapped off and the remaining liquid was allowed to drain. The neutralized supernatant (280 μL) from above was then added to the ion exchange columns. The ^3^H_2_O was eluted by adding 1.7-mL deionized water (two washes with 850 μL) and collecting the flow directly into scintillation vials. The collected elute was mixed with 5 mL of Ultima Gold scintillation fluid. To decrease batch-to-batch variability, radioactivity (RDA) vials (i.e., positive control) were prepared by mixing 20 μL of the reaction mixture (^3^H-palmitate/BSA/carnitine) with 1.5 mL of deionized water and then mixing with 5 mL of scintillation fluid. Levels of radioactivity were measured as counts per minute (CPM) by a scintillation counter.

Palmitate oxidation rates were expressed as nmol of [9,10-^3^H]palmitate oxidized per hour per milligram of protein. This was calculated by subtracting the CPM of samples from that of the blank wells (Δ), which was then divided by the RDA CPM. This number was then multiplied by 1,429 [256.4 (molecular weight of palmitate)/0.062 mM concentration in reaction mixture]. The product was multiplied by 500 [×1,000 to convert from μg to mg divided by 2 (h)]. Finally, this was normalized to protein content per well [see below for the calculations (21)]:

$$\left.\text{Results}\;({\text{nmol}}^{3}H\text{-FA(fatty}\;\text{acid})/h/mg\;\text{protein}\right)\;\;=((\Delta CPM/\text{RDA}\;\times 1,429)\times 1/\mu g\;\text{protein}\;\times 500)$$

ΔCPM=CPMsample-CPMblank

RDA=CPMofRDAsample

μgprotein=determinedbyBCAassay

### Immunoblots

Kidney cortices were homogenized in lysis buffer [150 mM NaCl, 50 mM Tris-HCl, 1 mM EDTA and 2% SDS with protease and phosphatase 2/3 inhibitors (1:100) (P8340, P5726, P0044, Sigma)]. Lysates were centrifuged and reduced with dithiothreitol (DTT, 1:100) and quantified using BCA protein kit. Lysates were separated using SDS-PAGE and transferred onto nitrocellulose membranes, blocked with 5% milk, followed by incubation with primary antibodies for total OXPHOS antibody cocktail (Abcam, ab110413), CPT1A (Abcam, ab128568), and β-actin (Cell Signaling, 8H10D10) overnight at 4°C. Immunoblots were incubated with the appropriate HRP-conjugated secondary antibody, imaged on Chemidoc (Biorad Chemidoc MP), and quantified by ImageJ.

### Quantitative PCR

Renal cortices were incubated in Lysis Matrix Tubes (MP Biomedicals) containing RLT buffer (Qiagen, 74106) with 2-mercaptoethanol (1:100, M3148, Sigma) before being purified on the RNeasy spin columns. RNA was extracted using the QIAGEN RNeasy Mini Kit, and the cDNA was generated using Bio-Rad’s iScript cDNA Synthesis Kit. The QuantStudio 6 Flex Real-Time system (Thermo Fisher Scientific, 4485691) was used to perform the quantitative PCR (qPCR) with 100-ng cDNA and iQ SYBR Green Supermix. The ΔΔCT equation was used to determine the relative mRNA expression with β-actin used as a reference gene. The primers used are listed in Table 5.

### Data Analysis and Statistics

GraphPad Prism 9 (GraphPad Software, San Diego, CA) was used to plot graphs and perform all statistics. One-way ANOVA and two-tailed Student’s *t* test were used for multiple comparisons and two-set comparisons, respectively. All data were expressed as mean ± SD. *P* < 0.05 was considered statistically significant.

### Affiliation with Washington University Kidney O’Brien Center’s Metabolism Core

The Seahorse bioflux analyzer and radioactive-labeled substrate oxidation assays are performed by the faculty of the Metabolism Core (who are all co-authors). These studies can be performed for kidney investigators by the Washington University Kidney O’Brien Center’s Metabolism Core (https://sites.wustl.edu/obrienckdcenter/home-2/metabolism-core/).

## RESULTS

### Immortalized Proximal Tubule Cells Are Less Oxidative than Primary Proximal Tubule Cells

The Seahorse assay is valuable for measuring mitochondrial respiration in adherent cells through detecting oxygen consumption rate (OCR). Generating these data using Agilent’s Mito Stress assay is straightforward, but several variables are important to produce valid and reproducible results. Achieving an equal number of cells per well/sample is important for valid results, and it is also important to optimize the cell number and concentration of reagents (e.g., FCCP) for a given reagent. We determined that 63k and 100k were ideal numbers of cells to plate for 96- and 24-well plates, respectively (Supplemental Fig. S1, A and B). Based upon a dose response for FCCP (Supplemental Fig. S1C), 2 μM was chosen for the primary proximal tubule-enriched cells used in our studies. Another variable that can impact the data is the normalization of cell number or protein content after the assay has been performed. This is crucial because cells are plated the day before the experiment. Depending upon the variable being tested, cells may grow differently over time, leading to more cells in one condition, or may adhere differently and detach during the assay. Normalization to cell number or protein content is both acceptable, although it is important to note that some conditions (e.g., cell cycle inhibitor) may affect cell size and impact cell number differently from protein expression. The key is that assessment of cell number (ideal) or protein content should be performed on samples after completion of the Seahorse assay.

**Figure 2.**
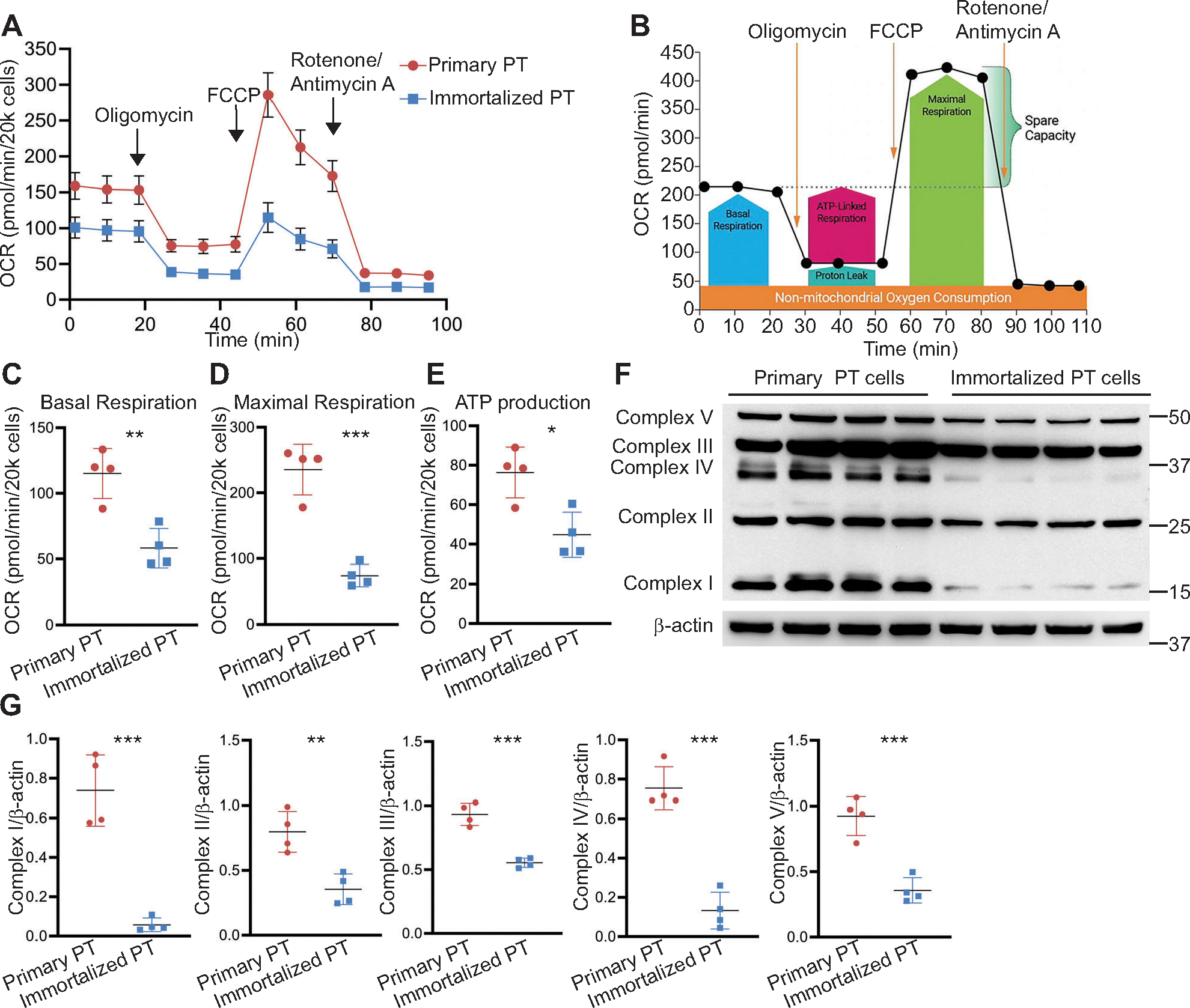
Immortalized proximal tubule cells are less oxidative than primary proximal tubule cells. *A*: representative data from one Mito Stress assay using the Agilent Seahorse XFe24 bioflux analyzer showing oxygen consumption rate (OCR) in primary proximal tubule (PT) cells and immortalized proximal tubule (immortalized PT) cells after treatment with oligomycin, FCCP, and rotenone/antimycin A. *B*: Agilent Seahorse Mito Stress test profile is shown (adapted from Agilent Seahorse XF Mito Stress Kit User Guide). Average basal respiration (*C*), maximal respiration (*D*), and ATP production (*E*) were significantly reduced in immortalized PT cells compared with primary PT cells (*n* = 4/group). Mitochondrial complexes were measured using immunoblots on cell lysates from immortalized and primary PT cells (*F*) and quantified (*G*). Results are reported as means ± SD, *P < 0.05, **P < 0.01, ***P < 0.001. Statistical significance between the two groups was determined by unpaired *t* test.

Immortalized cells are commonly used in research as they are more readily available than generating primary cell populations. Cells lose their oxidative respiratory capacity over time in culture, but the extent to which this occurs is unclear (22–24). Therefore, we directly compared oxidative respiration in an immortalized PT-cell population (see MATERIALS AND METHODS) (13) to that of freshly isolated primary PT cells, previously characterized (11, 12). The workflow of cell preparation, type of media used, etc. for the Seahorse bioflux analyzer is outlined in Fig. 1. The oxidative capacity of immortalized PT cells was significantly reduced compared with freshly prepared primary PT cells using the Mito Stress assay (Fig. 2, A and B). The basal respiration rate in primary PT cells was approximately twice as high as that from immortalized PT cells (Fig. 2C). Maximal respiration, the oxygen consumption rate (OCR) after disruption of the mitochondrial proton gradient by FCCP, was approximately threefold higher in primary PT cells compared with immortalized PT cells (Fig. 2D). Consistently, ATP production was also reduced in immortalized compared with primary PT cells (Fig. 2E). One potential explanation for the significantly reduced mitochondrial respiration in immortalized cells is a decrease in mitochondrial content. We detected a significant decrease in the expression of all mitochondrial complexes in immortalized compared with primary PT cells (Fig. 2, F and G). Taken together, these data indicate higher mitochondrial activity in primary cells compared with immortalized PT cells, likely due to preserved mitochondrial complex expression. Therefore, primary cells are more useful than immortalized PT cells for the measurement of oxidative metabolism.

### Primary Cells Lose Their Oxidative Ability with Time in Culture

**Figure 3.**
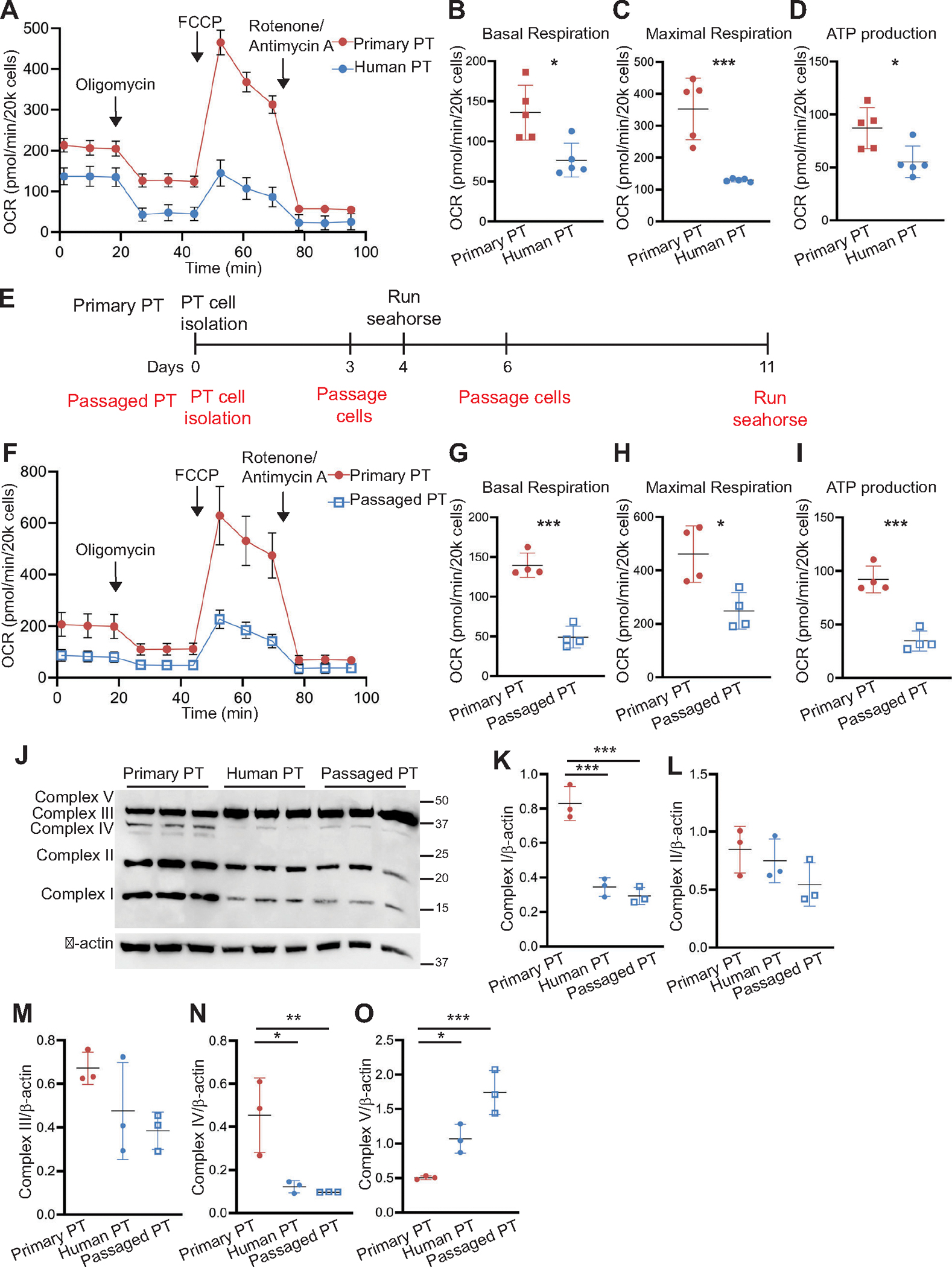
Primary cells lose their oxidative capacity with time in culture. *A*: representative Mito Stress assay using Seahorse XFe24 bioflux analysis shows oxygen consumption rate (OCR) in human primary tubule cells and primary proximal tubule (PT) cells. Basal respiration, maximal respiration, and ATP production (*B*–*D*) from five different experiments with human primary PT cells and freshly isolated PT cells. *E*: timeline showing the different number of days in culture and passages of primary PT cells from those primary cells kept longer (passaged PT cells). *F:* representative Mito Stress assay showing OCR of primary PT cells and cells grown longer in culture (passaged PT). Basal respiration, maximal respiration, and ATP production from primary PT cells and passaged PT cells are shown, *n* = 4 separate experiments (*G*–*I*). Immunoblots of PT cell lysates showing mitochondrial complexes (*J*) with quantification of complexes I–V normalized to β-actin, *n* = 3 separate experiments (*K*–*O*). Results are reported as means ± SD, *P < 0.05, **P < 0.01, ***P < 0.001. Statistical significance between the two groups was determined by an unpaired *t* test. One-way ANOVA was performed followed by Sidak’s multiple comparison for (*K*–*O*).

Immortalized cells have lower oxidative capacity compared with primary cells, but immortalized cells have often been stored for years and passaged several times. This raises the question: do all primary cells have equal oxidative capacity? Commercially available primary human tubular cells are cryopreserved and have been passaged before usage. We compared the oxidative capacity of cryopreserved human primary PT cells from Lonza with freshly isolated murine PT cells. After an initial passage (see MATERIALS AND METHODS), we plated freshly thawed human PT cells directly onto plates for bioflux analysis by Seahorse. The basal and maximal respiration as well as ATP production in primary human PT cells were significantly lower than those of freshly isolated murine primary PT cells (Fig. 3, A–D). The differences in oxidative capacity could be due to inherent differences between species (human vs. murine) or time in culture. To define the role of time in culture, we compared the oxidative capacity of primary PT cells grown in culture for 4 versus 11 days (Fig. 3E). The primary PT cells that had been grown in culture longer (passaged PT) had significantly reduced basal respiration, maximal respiration, and ATP production compared with those in culture for a shorter time (Fig. 3, F–L). Consistent with the OCR data, the expression of most mitochondrial complexes was suppressed in human PT cells and passaged PT cells compared with primary murine PT cells in culture for a shorter time (Fig. 3, J–O). These data indicate that primary PT cells exhibit reduced oxidative capacity with increased time in culture, potentially due to reduced mitochondrial complex expression. Thus, the time that primary cells are in culture is an important variable that impacts data obtained from Seahorse bioflux analyses.

### Mitochondrial Oxidative Respiration Is Higher in Tubules Ex Vivo Compared with Primary Proximal Tubule Cells

**Figure 4.**
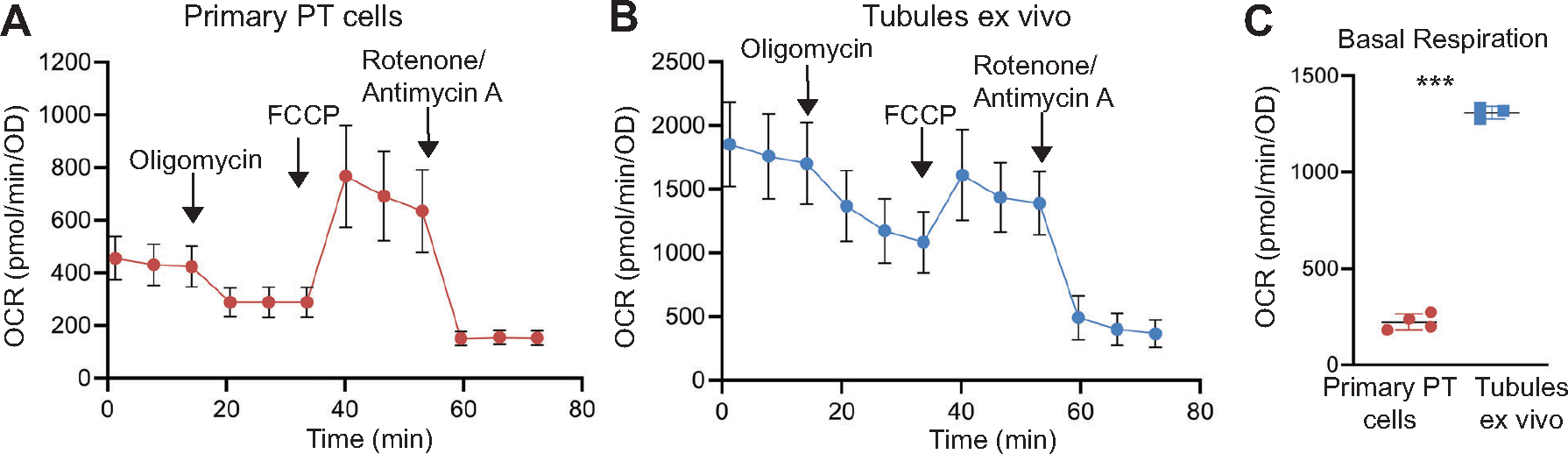
Tubules ex vivo have greater oxidative capacity than primary proximal tubule (PT) cells. Mito Stress assay performed by Seahorse XFe96 bioflux analyzer shows oxygen consumption rates (OCR) for primary PT cells (*A*) and freshly isolated tubules (*B*). *C:* Average of basal respiration of primary PT cells (*n* = 4 mice) and tubules ex vivo (*n* = 3 mice). Results shown as means ± SD, ***P < 0.001. Statistical significance was determined by unpaired *t* test.

Plated cells, even primary cells that have been freshly isolated, have been removed from their normal three-dimensional environment and are growing on stiff plastic rather than a biological basement membrane. Although the Seahorse bioflux analyzer is primarily intended for adherent cells, others have adapted this assay for tissue ex vivo. Therefore, we compared the mitochondrial respiration of freshly isolated tubules ex vivo with that of primary PT cells. We generated kidney tubules ex vivo using a previously published protocol with slight modifications (see MATERIALS AND METHODS, Fig. 1) (17). One challenge with using tubules ex vivo is that you cannot normalize to cell number, so we used the SRB assay as a measurement of protein content (see MATERIALS AND METHODS and Fig. 1). Another difficulty is the adhesion of these tubules during the Seahorse assay, which requires mixing of the injected compounds. After a trial with several different coating materials, we used poly-d-lysine to facilitate better adhesion (Supplemental Fig. S2, E and F). Tubules ex vivo had significantly higher OCR compared with primary PT cells (Fig. 4, A–C). However, a gradual decline in OCR over time was observed, which we attribute to reduced tubular adhesion rather than depletion of oxygen in the media (Supplemental Fig. S3, A–D).

**Figure 5.**
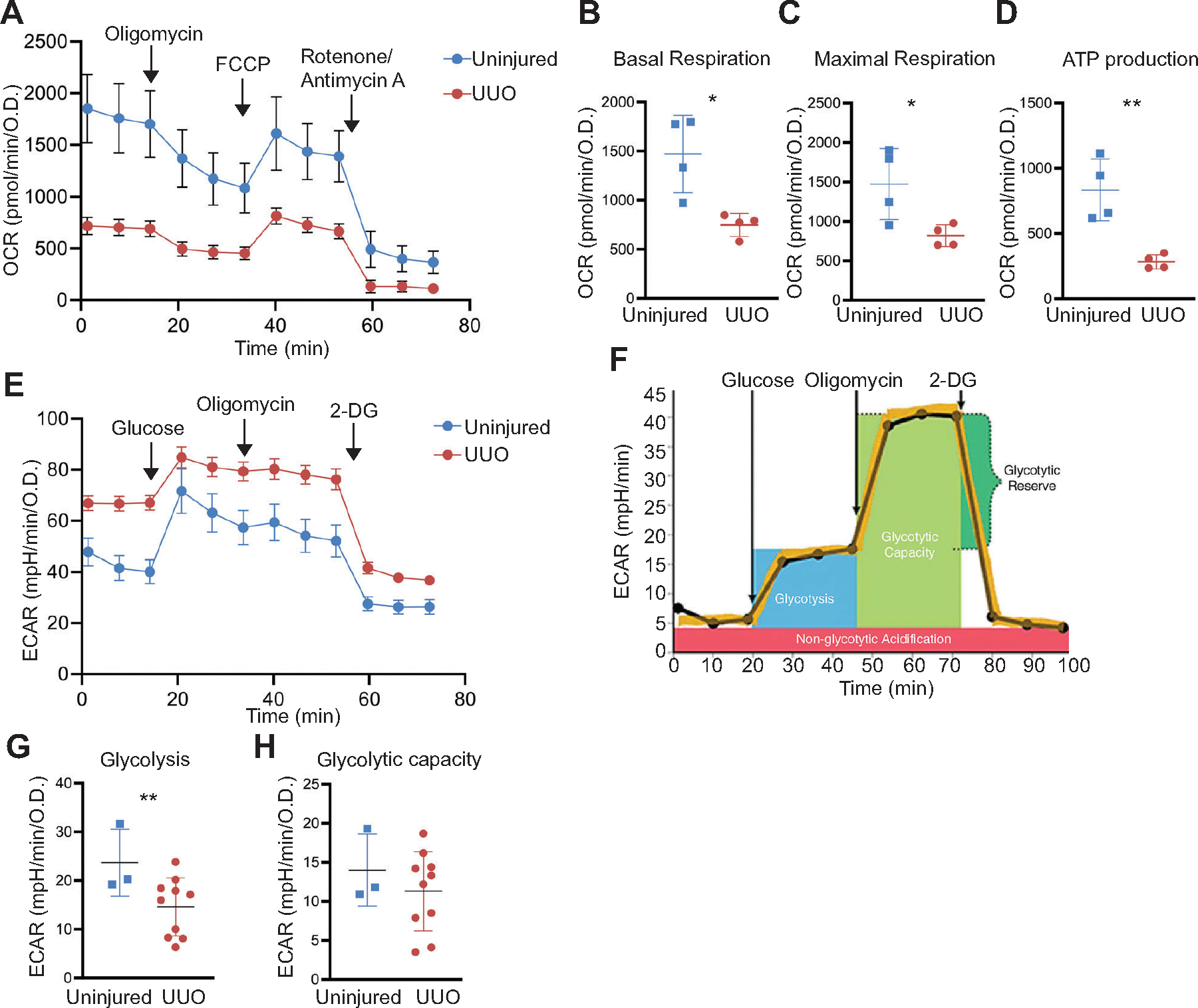
Metabolic changes in tubules ex vivo after unilateral ureteral obstruction (UUO)-induced injury. A representative tracing of oxygen consumption rate (OCR), measured by Seahorse XFe96 bioflux analyzer, in tubules ex vivo isolated from uninjured and obstructed kidneys (2 days after UUO) (*A*). Basal respiration, maximal respiration, and ATP production in tubules ex vivo from four separate experiments are shown (*B*–*D*). Glycolysis was measured in tubules ex vivo from uninjured kidneys (*n* = 3) and those 2 days after UUO (*n* = 10) using extracellular acidification rate (ECAR) per Agilent’s glycolysis assay (*E* and *F*). Glycolysis (*G*) and glycolytic capacity (*H*) were calculated, and all results are reported as means ± SD, *P < 0.05, **P < 0.01. Statistical significance was determined by an unpaired *t* test.

In both acute and chronic kidney injury, metabolism is altered. We assessed OCR and glycolysis, by measuring extracellular acidification rate (ECAR), using the Seahorse bioflux analyzer in tubules ex vivo from uninjured kidneys compared to those injured by unilateral ureteral obstruction (UUO). In the chronically injured kidney, fibrosis may complicate the process of isolating tubules, so we used kidneys 2 days after induction of UUO. The oxidative respiration of tubules from injured kidneys and ATP generation were reduced significantly compared with tubules isolated from healthy kidneys (Fig. 5, A–D). A shift to glycolysis has been described in injured tubules (25). Although basal ECAR was higher in the injured tubules, glycolysis (ECAR in response to glucose) was slightly decreased in the injured tubules with no significant change in glycolytic capacity (Fig. 5, E–H). Thus, Seahorse bioflux analysis on kidney tubules ex vivo revealed significantly reduced mitochondrial respiration following kidney injury.

### Fatty Acid Oxidation Assay in Tissue Ex Vivo

**Figure 6.**
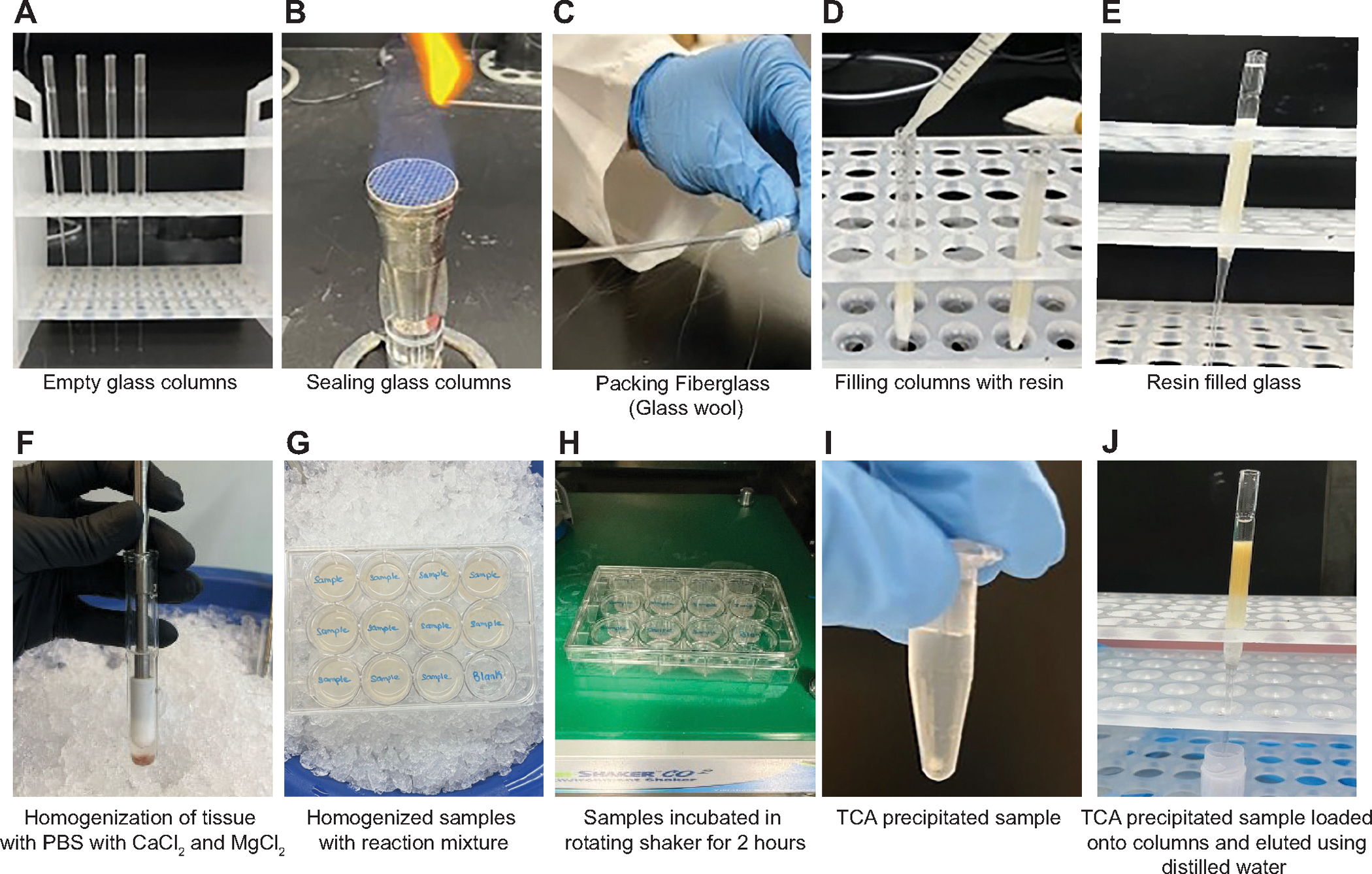
Radioactive fatty acid oxidation (FAO) assay workflow. The day before performing the FAO assay (*A*), seal the Pasteur Pipet tips by melting them with a Bunsen burner (*B*) and plug the pipets with 1 cm fiberglass (*C*). *D*: fill the sealed columns with 2.4 mL of resin slurry. Carefully remove the air bubbles and let the resin settle overnight at 4°C (*E*). On the day of the assay, homogenize the freshly isolated kidney cortex in PBS plus CaCl_2_/MgCl_2_ (*F*). Homogenization step should be performed gently on ice using a Dounce homogenizer (up and down 15×, possibly more for fibrotic tissue) until all the tissue is homogenized. Add the homogenized samples into a 12-well plate and mix with ^3^H-palmitate reaction mixture (^3^H-palmitate/BSA/carnitine) (*G*). Make sure to include blank well (no kidney sample only PBS with the reaction mixture). Incubate the plate on a rotating shaker (50 rpm) at 37°C in a cell culture incubator for 2 h (*H*). Then transfer 150 μL of each sample into a new tube containing 150 μL of 10% trichloroacetic acid (TCA). *I*: vortex the samples and centrifuge for 10 min at 2,200 *g* at 4°C to pellet the precipitated unmetabolized palmitate. Transfer the supernatant to a new tube with 46 μL of 6 M NaOH. Snap the sealed tip of the columns and allow the remaining liquid to drain completely. Place a scintillation vial at the tip of each column and slowly add the samples to the columns followed by 1.7 mL of deionized water. Make sure to add the deionized water slowly not to disrupt the resin. Collect the flow through directly into the vials (*J*). Allow the columns to drain completely into the vials and add 5 mL of Ultima Gold scintillation fluid into each collected elute and vortex well. Prepare separate radioactivity vials (RDA, positive controls) by mixing 20 μL of the reaction mixture with 1.5 mL of deionized water and 5 mL of scintillation fluid. Measure radioactivity as counts per minutes using a scintillation counter. Collect the remaining tissue homogenate from each well and determine protein concentration using BCA assay. Calculate the palmitate oxidation rates per hour per milligrams of protein (see MATERIALS AND METHODS for equation).

**Figure 7.**
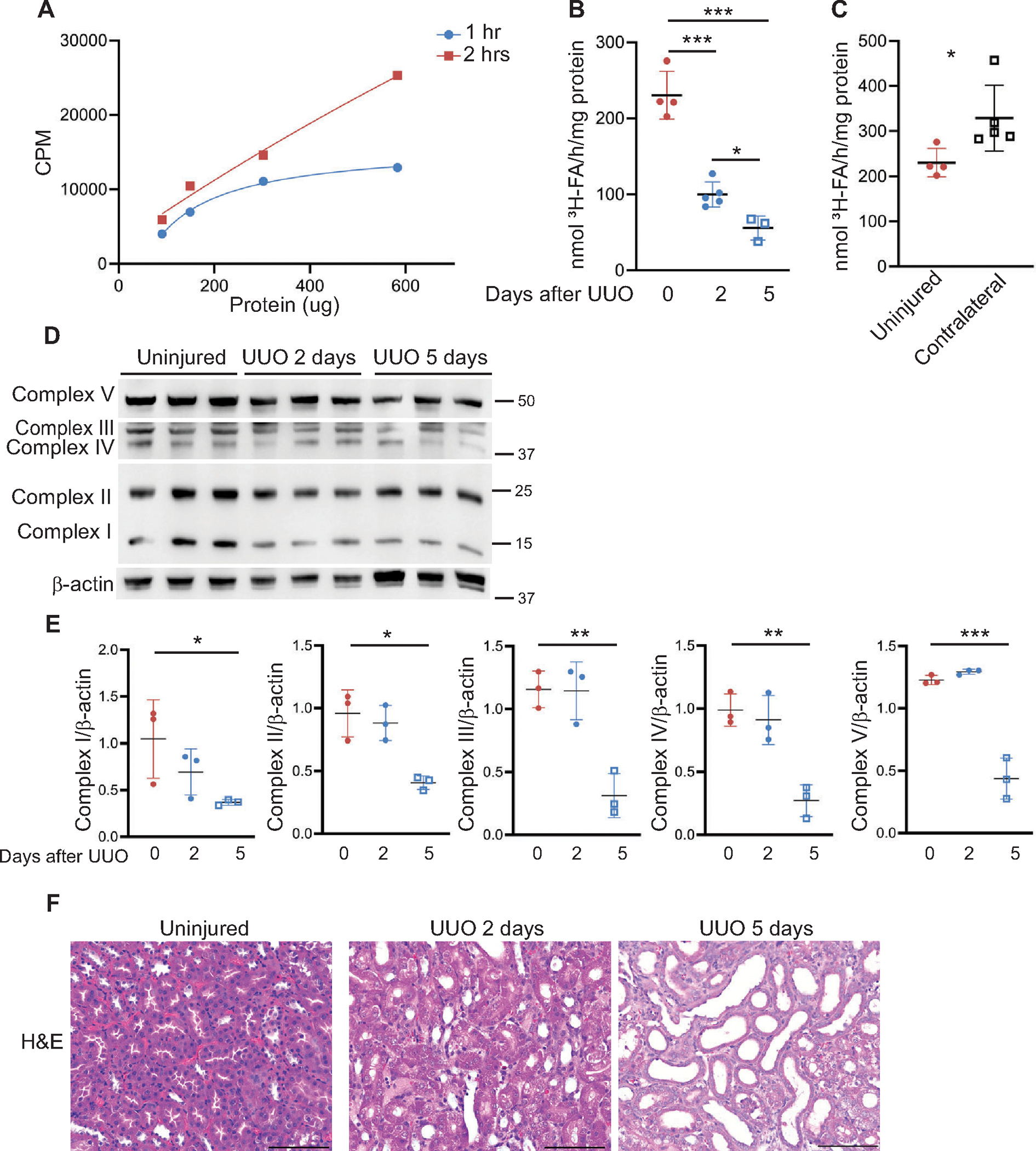
^3^H-palmitate oxidation assay shows decreased fatty acid oxidation at early time points after obstruction. *A*: dilutions of uninjured kidney cortical tissue were incubated with ^3^H-palmitate, a long-chain fatty acid, for 1 or 2 h and radioactivity measured in counts per minute (CPM). *B*: palmitate oxidation measured in kidneys from uninjured (*n* = 4), 2-day (*n* = 5) UUO-, or 5-day (*n* = 3) UUO-injured mice and expressed as nmol ^3^H-FA (fatty acid)/h/mg protein with each dot indicating a separate kidney. *C*: palmitate oxidation rates were measured from uninjured kidneys and the contralateral kidneys from 5-day UUO-injured mice. *D* and *E*: immunoblots assessed the expression of mitochondrial complexes in kidney cortices from uninjured, UUO, 2D (2 days), and UUO, 5D (5 days) mice with β-actin as a loading control and quantified using Image J (*n* = 3 kidneys per group). *F*: hematoxylin and eosin staining of uninjured and injured kidneys is shown. Results are shown as means ± SD, *P < 0.05, **P < 0.01, ***P < 0.001. Unpaired *t* test was used for *C*, and one-way ANOVA was performed followed by Sidak’s multiple comparisons for all others. UUO, unilateral ureteral obstruction.

Tubules ex vivo have increased mitochondrial respiration compared with cultured cells, but the gradual decrement of respiration over time (Figs. 4B and 5A) likely reflects the loss of adherence by some tubules to the bottom of the dish (Supplemental Fig. S3). Another approach to measure metabolism in tissue ex vivo is through radioactive isotope-labeled substrate (e.g., pyruvate or fatty acid) oxidation assays. Proximal tubule cells, comprising most of the kidney cortex, preferentially use long-chain fatty acids for β-oxidation to generate acetyl-CoA for use in the TCA cycle. To measure fatty acid oxidation (FAO) in the kidney tissue, albumin-bound ^3^H-labeled palmitate is incubated with kidney cortical tissue as demonstrated in Fig. 6. If the palmitate is fully metabolized, the ^3^H will label the H_2_O produced by the electron transport chain during the oxidation of reducing equivalents (e.g., NADH) generated in the TCA cycle. ^3^H_2_O can then be quantified by a scintillation counter, and the radioactivity (i.e., oxidized ^3^H-palmitate to ^3^H_2_O) can be converted into measurements of fatty acid oxidation/h/mg (see MATERIALS AND METHODS). Different amounts of kidney protein (100–600 μg) and incubation durations (1 and 2 h) were used to optimize the conditions (Fig. 7A). Incubation with ^3^H-labeled palmitate for 2 h was selected for future studies based on the linear increase in CPM with increasing amounts of protein. Less than 5% of the total palmitate provided was oxidized, so the availability of palmitate is not rate-limiting.

We then measured how FAO changes at different times after UUO injury (2 and 5 days). Injured kidneys, as expected, had significantly lower amounts of palmitate oxidation compared with uninjured kidneys (Fig. 7B). The FAO rates of uninjured kidneys were compared with the contralateral kidneys of UUO-injured mice (Fig. 7C). The increased palmitate oxidation in the contralateral kidneys suggest a compensatory increase in metabolic activity in response to the obstructed kidney. Consistent with diminished rates of palmitate oxidation, the mitochondrial complex expression was significantly decreased in the 5-day UUO compared with uninjured kidneys (Fig. 7, D and E). There was a significant drop in respiration in obstructed kidneys after 2 days despite no significant difference in mitochondrial complexes compared with the uninjured kidneys. Histological changes were mild at 2 versus 5 days after UUO (Fig. 7F). In summary, we show a time-dependent decrease in fatty acid oxidation using this ^3^H-palmitate oxidation assay ex vivo with significant changes as early as 2 days after obstruction.

**Figure 8.**
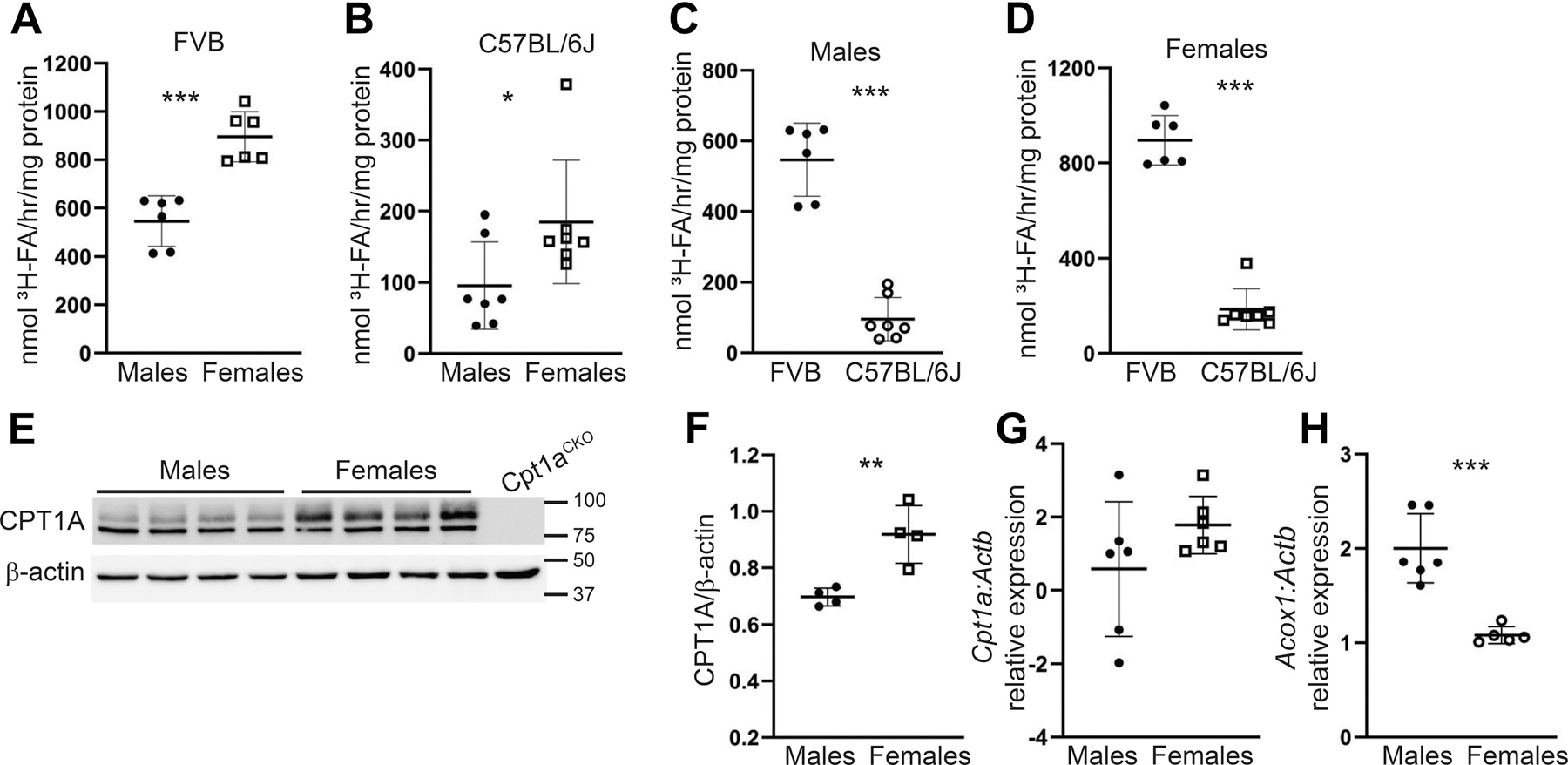
Sex- and strain-specific differences in kidney fatty acid metabolism. *A*–*D*: fatty acid oxidation was measured using ^3^H-palmitate in kidney tissue of uninjured male and female FVB (*n* = 6 mice per sex) and C57BL/6J mice (*n* = 7 mice per sex). Immunoblots of FVB kidney cortices show CPT1A protein expression with a *Cpt1a* conditional knockout kidney (*Cpt1a*^CKO^) as negative control (*E*). CPT1A protein expression, normalized to β-actin, was quantified (*F*). *G* and *H*: gene expression of *Cpt1a* and *Acox1*, normalized to β-actin (*Actb*) are shown. Results are reported as means ± SD, *P < 0.05, **P < 0.01, ***P <0.001. Unpaired *t* test was used for statistical significance.

Sex-specific differences in metabolism have been described in other organs, and we investigated sex-specific differences in kidneys using the ^3^H-palmitate oxidation assay. Kidney cortices from uninjured female FVB and C57BL/6J mice had significantly higher rates of palmitate oxidation compared with those from males (Fig. 8, A and B) without differences in mitochondrial complex expression (Supplemental Fig. S4). FAO also differed by strain with FVB mice having significantly higher FAO compared with C57BL/6J mice regardless of sex (Fig. 8, C and D). To better understand the sex-specific differences, we measured protein and gene expression of carnitine palmitoyltransferase 1 A (CPT1A, *Cpt1a*) and acyl-CoA oxidase 1 (ACOX1, *Acox1*), the rate-limiting enzymes of mitochondrial and peroxisomal FAO, respectively (26, 27). Although no sex-specific differences were noted in *Cpt1a* gene expression, females had significantly increased CPT1A protein expression compared with male kidneys (Fig. 8, E–G). By contrast, male kidneys had significantly increased *Acox1* gene expression compared with females (Fig. 8H). Thus, our ^3^H-palmitate oxidation assay revealed a significant increase in long-chain fatty acid oxidation in female compared with male mice across two different strains, a finding that may be explained by differences in CPT1A protein expression.

## DISCUSSION

The kidney field needs cost-effective and accessible functional metabolic assays that can validate important changes in the expression of metabolism-related genes identified by robust transcriptomics techniques. Stable isotope tracing studies that reveal dynamic pathway changes are the gold standard, but these are very expensive and require a high level of bioinformatics expertise. The Seahorse bioflux analyzer, available in most institutional Core facilities, and substrate oxidation assays are two functional metabolic assays that are more cost-effective and user friendly for most investigators. Although Agilent provides detailed protocols for using the Seahorse bioflux analyzer, there is little data describing how the choice of cells being used (primary vs. immortalized) or time in culture affects mitochondrial respiration. We describe how these factors impact OCR, raising important questions about critical methodological information that is often omitted from articles regarding Seahorse data and that can greatly affect reproducibility. In addition, we use a ^3^H-palmitate assay to measure fatty acid oxidation ex vivo, which, to our knowledge, has not been previously used in the kidney. This assay showed a time-dependent decrease in fatty acid oxidation in kidneys after UUO and identified sex-specific and strain-specific differences in FAO in uninjured kidneys.

Our Seahorse bioflux data reveal the importance of cell type when defining mitochondrial respiration. Although immortalized cell lines/populations are widely available, oxidative respiration is significantly suppressed in an immortalized PT population compared with primary PT cells, likely due to mitochondrial dropout. Others have reported variable fidelity of transcripts between different PT cells lines compared with transcriptomic profiling of the native proximal tubule (7). Although our studies did not examine PT cell lines from other species (e.g., opossum, rat), the functional metabolic differences between primary cells and cell lines are likely to be greater than the differences between cell lines given the time in culture. One limitation of our studies is that, while the primary PT, immortalized PT, and human PT cells were all assayed in the same media (SLM followed by KHB), they were initially grown in different media. The human PT cells were obtained commercially by Lonza and the recommended media for growth is proprietary. It is also possible that the immortalized PT cells still had some expression of the large T antigen at the time of assay, though we do not think this significantly impacted the data given the reduction in mitochondrial complexes. In addition, we cannot exclude the possibility that treatment of conditionally immortalized PT cells for 3 days with interferon-γ, shown to induce metabolic and mitochondrial changes, impacted the Seahorse data (28, 29). PT cells were free of interferon-γ for 24 h before Seahorse. The choice of cells (primary vs. immortalized, early vs. late passage) to be used by an investigator depends upon the metabolic question being asked. Immortalized cells may be a reasonable initial approach to use with Seahorse bioflux analysis to screen compounds for a certain metabolic response. However, our data underscore the importance of primary cells when investigating mitochondrial respiration given the significant differences in OCR compared with immortalized cells.

Even among primary cells, the duration in cell culture and number of passages also significantly affects mitochondrial respiration. Primary PT cells grown in culture for 11 days, P3, had significantly lower OCR compared with those in culture for 4 days, P1 (30, 31). Sometimes, there are legitimate reasons for needing to extend time in culture before Seahorse analysis. However, it is important that any comparisons are performed between cells in culture for the same length of time and passage number. Passage number is often listed as a range (e.g., P1–P3), but for primary cells being used for metabolic analysis, exact passage numbers and days in culture should be stated in published Methods to enhance reproducibility. Guidelines regarding the type of media are difficult as it may depend upon the specific metabolic question. However, in general, restricting the glucose to physiological levels or below is recommended. It is critical to determine the optimal number of cells for Seahorse plating as we did for proximal tubule cells (Supplemental Fig. S1). However, the optimal number may differ depending upon the cell type of interest, and the optimal number of other cell types (e.g., collecting duct cells) may differ.

Some investigators perform Seahorse assays on isolated mitochondria, which has the advantage of detecting specific mitochondrial protein defects and direct responses to potential treatments (32). Using isolated mitochondria can also provide better control over the availability of substrates and ADP compared with intact cell respiration (33). However, the disadvantages are that this approach does not replicate the cellular environment, so physiological relevance may be less, and mitochondria may be damaged in the isolation process (16).

Performing Seahorse on tubules ex vivo resulted in even higher rates of respiration compared with primary PT cells. Two days after UUO-induced injury, basal OCR in tubules ex vivo were reduced by ~50%. Unexpectedly, glycolysis was also reduced in tubules ex vivo from UUO kidneys despite higher basal levels of ECAR. Others have postulated a switch to glycolysis after injury and shown increased expression of enzymes important to glycolysis after injury (25). However, very few studies have examined functional glycolysis in injury. One potential explanation for the higher basal ECAR but lower levels of glycolysis (i.e., ECAR increase in response to glucose) is that the isolated tubules may have existing pyruvate or glucose (from gluconeogenesis) that was not added to the media to generate increased ECAR. Our data are consistent with our previous study showing all functional parameters of glycolysis were reduced in isolated tubules ex vivo after a septic model of acute kidney injury (17). The utilization of substrates upstream in the glycolytic pathway by other competing metabolic pathways may lead to lower functional glycolysis despite the increase in the expression of rate-limiting glycolytic enzymes.

Seahorse analysis using tubules ex vivo is a valuable tool to assess injury-induced changes in basal respiration. However, this method presents some challenges. First, plating tubules rather than individual cells makes equal plating difficult, although correcting for protein content can help. The bigger problem is that the Seahorse is really designed for adherent cells as the system will mix the media after each injection (e.g., oligomycin). Despite the use of poly-d-lysine and a centrifugation step, the OCR declined over the Seahorse assay. This was not due to oxygen depletion based on the raw oxygen levels in the media (Supplemental Fig. S3, A and B). However, it appeared that the number of adherent tubules had declined at the end of the assay versus the beginning (Supplemental Fig. S3, C and D). This lack of adhesion likely explains the gradual decline in OCR over time and lower-than-expected values for maximal respiration. To augment adhesion, we also coated the Seahorse plates with other substances (e.g., Cell-Tak, Matrigel), but without an improvement in tubule adhesion (Supplemental Fig. S2). Our data suggest that using tubules ex vivo for Seahorse bioflux analyses yields much higher levels of basal respiration than plated cells. However, subsequent measurements on tubules ex vivo, necessary to calculate maximal respiration and respiratory capacity, are less reliable than using plated cells.

Our studies used both the well-described Agilent Mito Stress and Glycolysis assays to test the metabolism of different types of cells. Agilent also has protocols for assessing substrate-specific metabolism such as FAO. One caveat is that etomoxir, an inhibitor of carnitine palmitoyltransferase (CPT), which is used to show FAO-dependent respiration, has off-target effects at concentrations above 5 μM (34). As etomoxir also inhibits complex I as an off-target effect, decreases in OCR observed with etomoxir may be due to effects on complex I and not due to FAO (35, 36). Thus, extrapolating rates of FAO based on the decrease in respiration in response to etomoxir is less accurate than measuring FAO by use of labeled substrate.

We report a method for assessing substrate-specific oxidation in tissue ex vivo using ^3^H-palmitate that is novel to the kidney. Others in the kidney field have performed similar measurements of fatty acid oxidation using ^14^C-palmitate (37). Our method has two advantages over the ^14^C-palmitate approach: *1*) ^14^C-palmitate requires the capture and quantification of ^14^CO_2_ that is more technically difficult and *2*) ^3^H-palmitate measures complete oxidation through the ETC compared with ^14^C-palmitate that only reflects oxidation in the TCA cycle. This method is relatively inexpensive and only requires a scintillation counter. The information obtained is similar to that of high-flux respirometry using Oroboros. An advantage of high-flux respirometry (Oroboros) is that it allows measurement of the activity of individual mitochondrial complexes in response to certain substrates. However, the Oroboros equipment is quite expensive, and its use requires specialized training. Also, Oroboros starts with a much smaller amount of tissue (e.g., 3–5 mg), compared with ~40 mg of tissue for substrate oxidation assays, so the latter better reflects whole organ metabolism and is less subject to sampling bias.

At 2 days after UUO, mitochondrial respiration from tubules ex vivo and fatty acid oxidation in tissue ex vivo was suppressed by ~50% using Seahorse and ^3^H-palmitate, respectively. Although complex I expression was reduced, expression of other mitochondrial complexes was still maintained 2 days after UUO, and most cortical tubule injury is relatively mild at this early stage. These assays suggest that reduced oxidative capacity, and specifically fatty acid oxidation, is an early event in the obstructed kidney. We also demonstrate that FAO in the contralateral kidney of obstructed mice is significantly higher than in uninjured kidneys, even when adjusted for protein content. The contralateral kidney in a UUO-injured mouse is often used as a control, but our studies show that this kidney is metabolically different from an uninjured kidney.

The ^3^H-palmitate assay showed a significant increase in fatty acid oxidation in female versus male kidney cortices from both FVB and C57BL/6J mice. Sex-dependent differences in kidney metabolism have been described (9, 38), but we are not aware of other studies showing sex-dependent differences in fatty acid oxidation in kidney tissue. Consistent with increased rates of FAO, female mice expressed more CPT1A, the rate-limiting enzyme for mitochondrial FAO (Fig. 6). Recently published data showed that female mice were protected against diabetic nephropathy, and this protection was dependent upon AMP-activated protein kinase (AMPK) activity (39). Similarly, female mice were protected from high-fat diet-induced renal dysfunction, and this was associated with higher levels of AMPK signaling (40). CPT1A is a well-described target of AMPK (41–43), and it is possible that increased AMPK/CPT1A pathway activity is responsible for the increased FAO in female mice, though additional studies are necessary to demonstrate a causal effect. The increase in *Acox1* gene expression noted in males has been previously reported by other groups (9). Our group and others have shown that ACOX1 and peroxisomal fatty acid oxidation may compensate when mitochondrial FAO is reduced (37, 44). It is possible that the increased *Acox1* gene expression in male compared with female mice is a mechanism to compensate for lower expression of CPT1A. In addition to sex-dependent differences in FAO, there were also significant differences in FAO between strains. FVB mice had almost 4× the oxidation rates compared with C57BL/6J mice regardless of sex. Such data indicate that the mouse strain must be considered when assessing metabolism, and comparisons across strains may be flawed.

The ability to reliably and reproducibly characterize metabolic changes in the kidney is important to further our understanding of how these metabolic alterations impact kidney injury. Seahorse bioflux analyzer is a commonly used metabolic assay to explore mitochondrial function and glycolysis. We present detailed protocols as well as data showing the impact of type of cells (primary vs. immortalized) and duration in culture on oxidative respiration. The advantages and disadvantages of using freshly isolated tubules ex vivo are also demonstrated using injured and uninjured kidneys. In addition, we show how substrate oxidation assays using ^3^H-palmitate, novel to the kidney, can be used to show early decreases in cortical FAO after UUO. This method is also used to demonstrate important differences in FAO between sexes and different strains of mice. The detailed protocols provided here and established differences between types of cells used, time in culture, female versus male sex, and strain are shown to enhance the accessibility of metabolic assays for the general kidney research community as well as the reproducibility of data.

## Supplementary Material

SUPPLEMENTAL MATERIAL

Supplemental Figs. S1–S4: https://doi.org/10.6084/m9.figshare.28582100.

Appendix

## Figures and Tables

**Table 1.** Supplements added to RPMI media

RPMI Media Additives	Concentration

Fetal bovine serum (FBS)	10%
Penicillin	100 IU/mL
Streptomycin	0.1 mg/mL
l-Glutamine	2 mM
*Proximal tubule cell supplements*	
Hydrocortisone	20 ng/mL
Insulin	4.2 μg/ml
Transferrin	3.8 μg/ml
Selenium	5 μg/mL
Triiodothyronine	6.68 ng/mL

**Table 2.** Supplements added to DMEM substrate limited media

Substrate Limited Media DMEM	Concentration

FBS	1%
Glucose	0.5 mM
Carnitine	0.5 mM
Glutamax	1 mM

**Table 3.** Krebs-Henseleit buffer components

Krebs–Henseleit Buffer (KHB)	Concentration

NaCl	111 mM
KCl	4.7 mM
CaCl_2_	1.25 mM
MgSO_4_	2 mM
NaH_2_PO_4_	1.2 mM
*Additives*	
Glucose	2.5 mM
Carnitine	0.5 mM
HEPES	5 mM
*pH 7.4*	

**Table 4.** Concentrations of palmitate and l-carnitine used in fatty acid oxidation assay

Items	Stock Concentration	Volume (per Sample)	RAD Reaction Mixture, μM	Final Concentration, μM

^3^H-palmitate	500 μM	25 μL	125	62
l-Carnitine	100 mM	1 μL	990	495
PBS (CaCl_2_ and MgCl_2_)		75 μL		

**Table 5.** List of primers

Gene Name	Forward	Reverse

*Cpt1a*	5′-CCATGAAGCCCTCAAACAGATC-3′	5′-ATCACACCCACCACCACGATA-3′
*Acox1*	5′-AGGGAATTTGGCATCGCAGA-3′	5′-CATGCCCAAGTGAAGGTCCA-3′
*Actb*	5′-GGGATGTTTGCTCCAACCAA-3′	5′-GCGCTTTTGACTCAGGATTTAA-3′

## Data Availability

Data will be made available upon reasonable request.

## NEW & NOTEWORTHY

We show significant metabolic differences between both primary and immortalized cells, and among primary cells with different durations in cell culture. In addition, ^3^H-palmitate oxidation in tissue ex vivo is described as a method novel to the kidney for assessing the complete oxidation of long-chain fatty acids. This method shows that female mice have significantly increased fatty acid oxidation across two different strains of mice and significant strain-specific effects on metabolism.
