# Supplementary material for: Assays to enhance metabolic phenotyping in the kidney
Source: Am J Physiol Renal Physiol. Author manuscript; Available in PMC 2025 Jun 8. (PMC12145867; doi:10.1152/ajprenal.00232.2024)
Supplement: Appendix [file NIHMS2081592-supplement-Appendix.pdf]

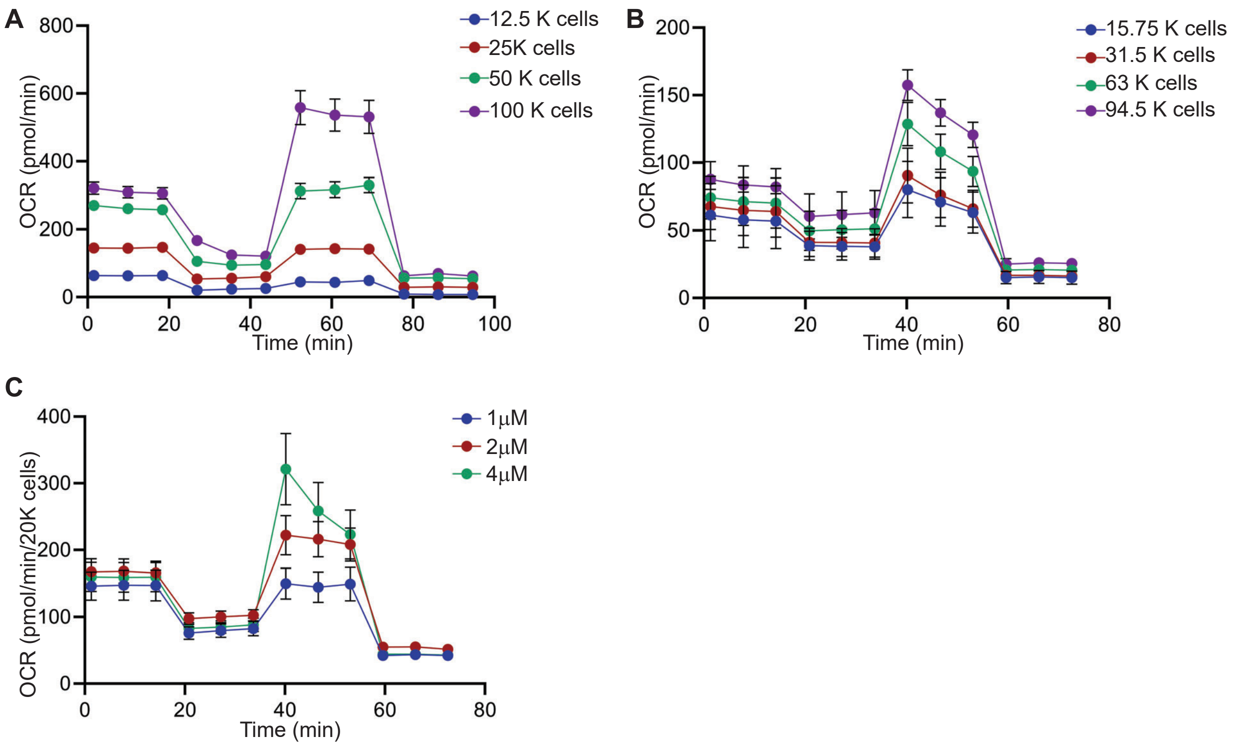

Appendix Figure 1: Representative Seahorse bioflux Mitostress assays showing oxygen consumption rate (OCR) of different number of plated cells on 24 well (A) and 96 well plates (B). (C) Representative Seahorse bioflux Mitostress assay showing OCR using different concentrations of FCCP (1, 2 and 4 μM).

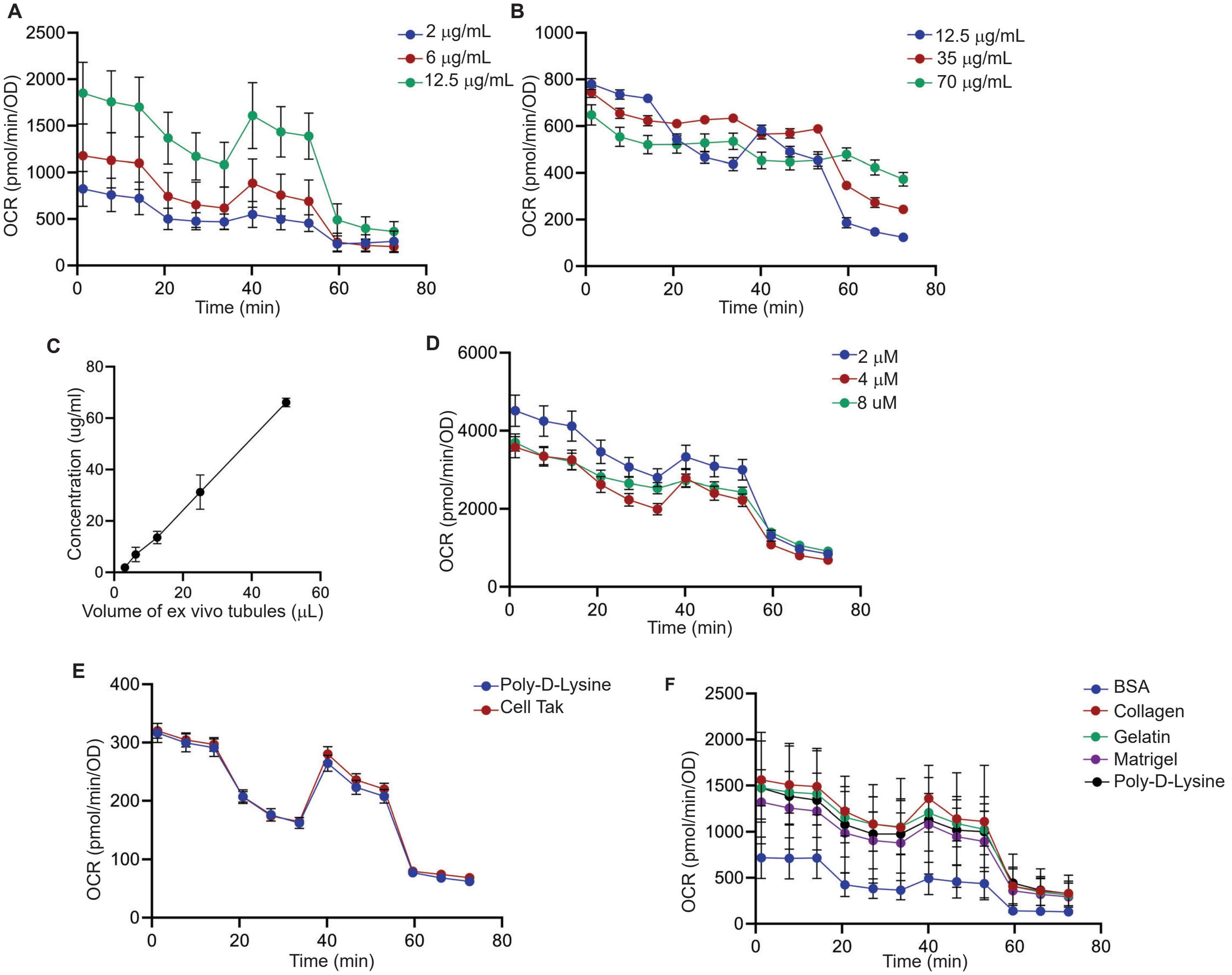

Appendix Figure 2: (A, B) Representative Mitostress on the Seahorse bioflux analyzer showing oxygen consumption rate (OCR) from different concentrations of freshly isolated tubules. (C) To help expedite plating, we plotted volumes of the tubules ex vivo (in 2 mL KHB) and determined a concentration curve that would allow us to plate an estimated concentration of 12.5 µg/mL based on volume. The normalization of OCR is based upon the sulforhodamine B (SRB) assay, performed on each well after the Seahorse run. Different concentrations of FCCP were used on tubules (D). (E) Mitostress assay was performed on freshly isolated tubules with either Poly-D-Lysine (50 µg/mL) or Cell-Tak (22.6 µg/mL) coated plates. (F) Seahorse bioflux assay showing OCR of freshly isolated tubules on either bovine serum albumin (BSA, 0.1%), collagen 1 (1:100), gelatin (0.1%), Matrigel (1:100) and Poly-D-Lysine (50 µg/mL) coated Seahorse bioflux plates.

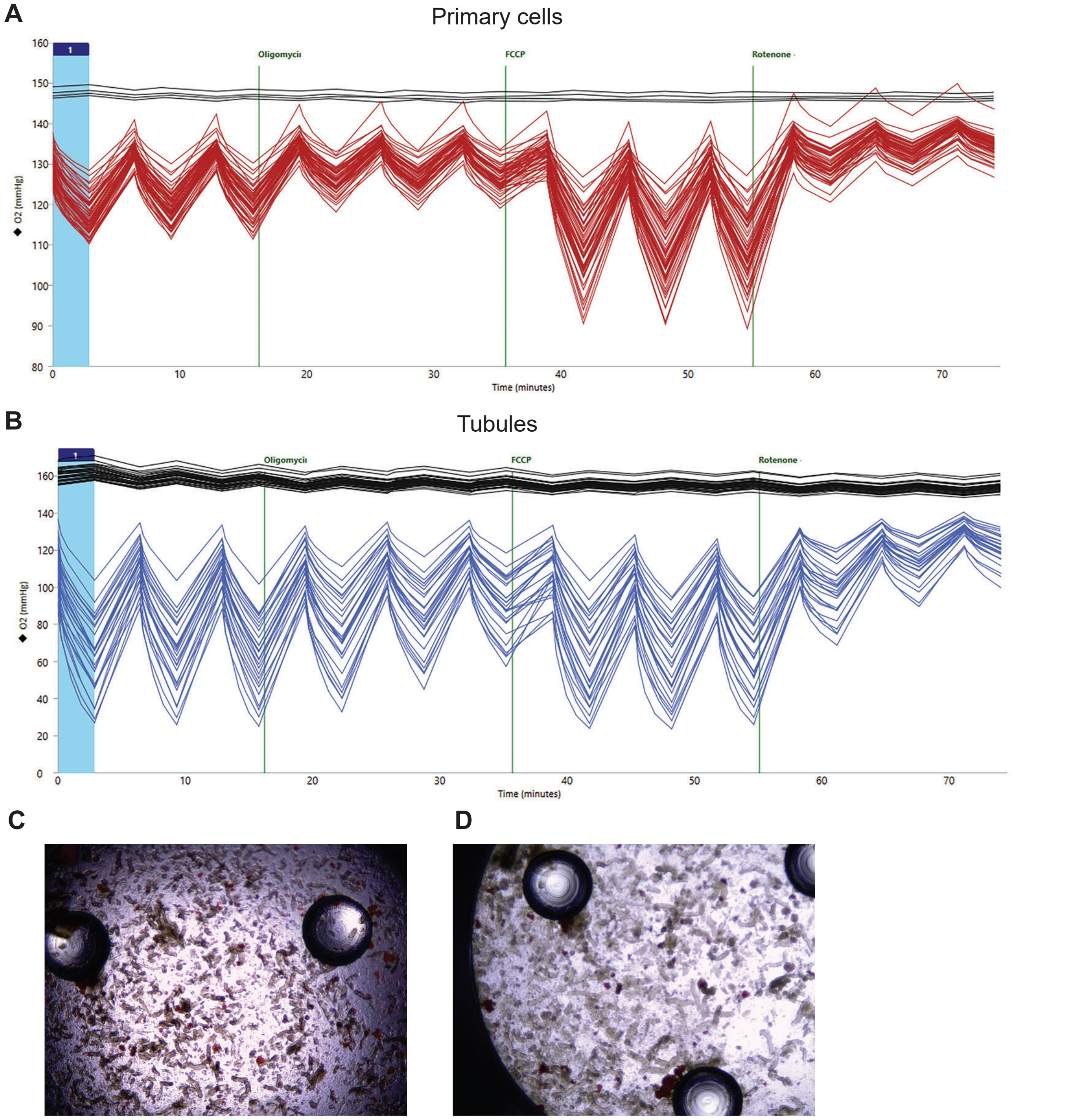

Appendix Figure 3: Representative Seahorse bioflux assay showing O_2 levels in mmHg during the MitoStress assay perfomed by Sehorse XFe96 bioflux analyzer for primary PT cells (A) and freshly isolated tubules (B). Pictures of the same 96 well with freshly isolated tubules plated before (C) and after (D) Seahorse assay.

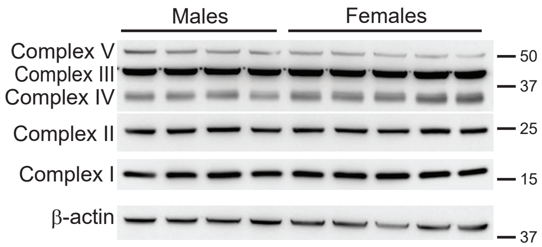

Appendix Figure 4: Kidney cortices immunoblotted for mitochondrial complexes from male and female kidneys.
